# Evaluation and Comparison of the Antibacterial Activity against *Streptococcus mutans* of Grape Seed Extract at Different Concentrations with Chlorhexidine Gluconate: An *in vitro* Study

**DOI:** 10.5005/jp-journals-10005-1360

**Published:** 2016-09-27

**Authors:** Milan Swadas, Bhavna Dave, Soham M Vyas, Nupur Shah

**Affiliations:** 1 Senior Lecturer, Department of Pedodontics and Preventive Dentistry Narsinhbhai Patel Dental College and Hospital, Visnagar Gujarat, India; 2 Professor and Head, Department of Pedodontics and Preventive Dentistry, KM Shah Dental College and Hospital, Sumadeep Vidyapeeth University, Vadodara, Gujarat, India; 3 Postgraduate Student, Department of Pedodontics and Preventive Dentistry, KM Shah Dental College and Hospital, Sumadeep Vidyapeeth University, Vadodara, Gujarat, India; 4 Postgraduate Student, Department of Pedodontics and Preventive Dentistry, KM Shah Dental College and Hospital, Sumadeep Vidyapeeth University, Vadodara, Gujarat, India

**Keywords:** Antimicrobial, Grape seed extract, *Streptococcus mutans.*

## Abstract

**Introduction:**

*Streptococcus mutans* has been implicated as primary microorganisms which cause dental caries in humans. There has been an increased interest in the therapeutic properties of some medicinal plants and natural compounds which have demonstrated antibacterial activities. Grape is one of the plants of this group which contains tannin and polyphenolic compound.

**Aim:**

To evaluate and compare antibacterial activity of grape seed extract at different concentrations with chlorhexidine gluconate against S. *mutans.*

**Materials and methods:**

Grape seeds were extracted with ethanol/water ratio of 70:30 volume/volume. The extracts were filtered through Whatman No. 1 filter paper until it becomes colorless. *Streptococcus mutans* strains were taken. To check the antimicrobial properties of grape seed extract at different concentration and chlorhexidine gluconate, they were added to S. *mutans* strain and incubated for 48 hours than colony-forming units/mL were checked.

**Results:**

Grape seed extract at higher concentration were found to be more potent against *S. mutans.* Chlorhexidine gluconate was found to have most potent antibacterial action compared to all different concentrations of grape seed extract.

**Conclusion:**

Grape seed extract as a natural antimicrobial compound has inhibitory effect against S. *mutans.*

**How to cite this article:**

Swadas M, Dave B, Vyas SM, Shah N. Evaluation and Comparison of the Antibacterial Activity against *Streptococcus mutans* of Grape Seed Extract at Different Concentrations with Chlorhexidine Gluconate: An *in vitro* Study. Int J Clin Pediatr Dent 2016;9(3):181-185.

## INTRODUCTION

Dental caries is a chronic infectious disease that produces widespread lesions throughout the world.^1^ It is a multifactorial disease in which there is interplay of three principle factors: The host (primarily the saliva and teeth), the mi-croflora, and the substrate or diet. In evaluating the caries risk of a patient, a number of factors must be taken into consideration. Salivary counts of mutans streptococci, combined with the measurement of salivary flow rate and buffer effect and sugar consumption, are frequently used for diagnostic and predictive purpose in cariology.^2^

*Mutans streptococci* (MS) group has been implicated as primary microorganisms which cause dental caries in humans and experimental animal models. Relying on sampling, this finding aims to isolate colony-forming units (CFU) from dental plaque and saliva.^3^ Salivary MS counts rarely exceed 10^7^ CFU/mL, and a highly significant correlation has been demonstrated between the salivary numbers of MS and caries prevalence.^4^

In the recent past, there has been an increased interest in the therapeutic properties of some medicinal plants and natural compounds which have demonstrated anti-cariogenic activities in both *in vitro* and *in vivo* conditions. Among these phytoconstituents, several polyphenolic compounds like tannins (catechins) and flavonoids seem to be the most promising biomolecules. Remarkable anticariogenic potency has been observed for alkaloids.^5^ Tannins are naturally occurring plant metabolites which can prevent bacterial dental plaque^6^ and can enhance remineralization of dental enamel.^7^ Grape is one of the plants of this group which contains tannin and poly-phenolic compound. So, grape seed extract also have remarkable antibacterial properties.^5^

Grape seed extract at lower concentration is ineffective against *S. mutans* bacterial strain.^8^ The purpose of this study was to check *in vitro* antibacterial effect of grape seed extract against *S. mutans* at different concentrations and with chlorhexidine gluconate.

**Fig. 1:**
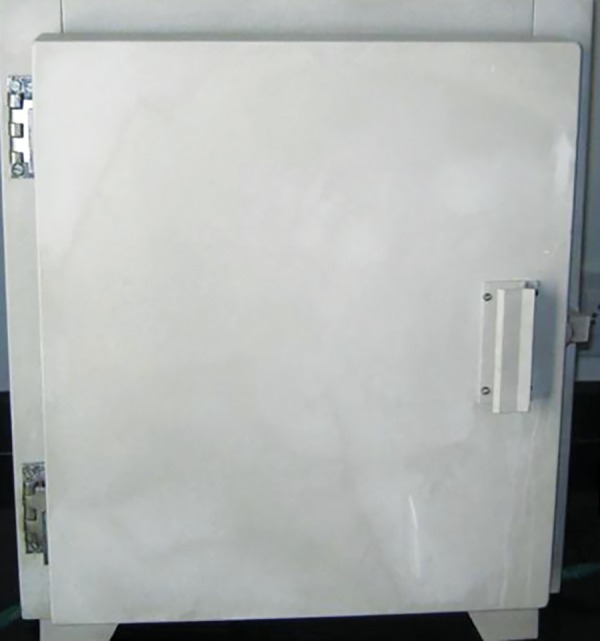
Incubator

## MATERIALS AND METHODS

The study was conducted in the Department of Paedo-dontics and Preventive Dentistry. Prior permission was taken from the Department of Microbiology of the university, and ethical approval was obtained from Ethics Committee of the University.

The following materials were used for the conduction of the study:

- Bacterial strains: *S. mutans* (ATCC 25175)
- Incubator (Fig. 1)
- Mitis salivarius bacitracin agar medium
- Disposable Petri dishes
- Inoculation loop for counting CFU.

### Procedure of preparing Grape Seed Extract (*Vitis vinifera L.)*

**Fig. 2:**
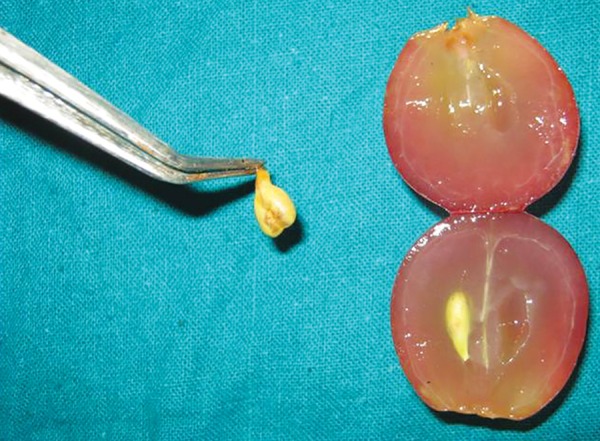
Grape seed

**Fig. 3:**
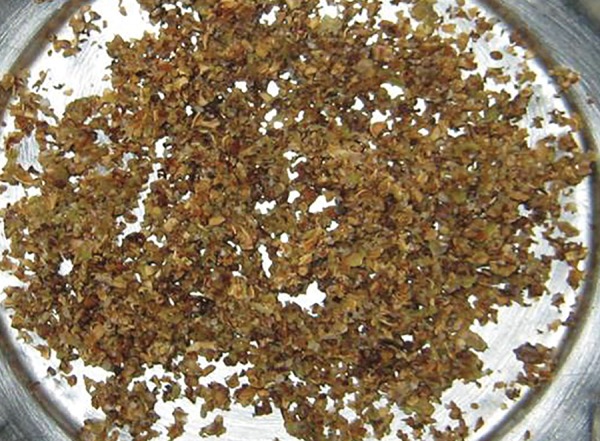
Grounded grape seed extract

Grape seeds were collected from red grapes which are available in market (Fig. 2). These seeds were dried under sunlight for 2 days, and then these seeds were grounded with cold pressure technique (Fig. 3). Ground grape seeds (100 gm) was extracted with ethanol/water ratio of 70:30, vol/vol, by maceration method under stirring at 45°C for 2 hours. The extract was filtered through Whatman No. 1 filter paper.^8^ It was sterilized by Millipore filter and final concentrations of grape seed extract were prepared. Grape seed extract was divided into three different experimental groups along with one positive control and one negative control group.

### Study Groups

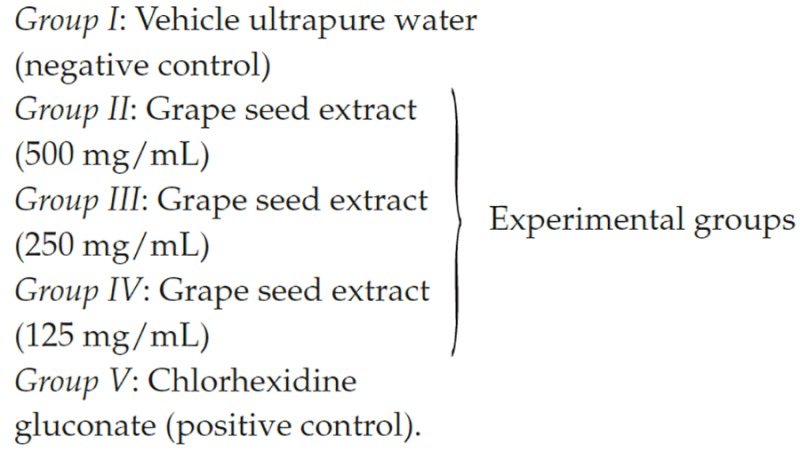



Thirty-five tubes (5 groups) were prepared containing test compounds dilutions and incubated at 37°C for 48 hours. Each concentration (500, 250, and 125 mg/mL) of the extracts was tested. The vehicle (ultrapure water) was used as negative control, and chlorhexidine gluconate was used as positive control as a 20% (w/v) aqueous solution. Each group has 7 Petri dishes. Numbering for these Petri dishes were done like I1 to I7 for the 1st group followed by other groups.

### Bacterial Strains and Growth Conditions

**Fig. 4:**
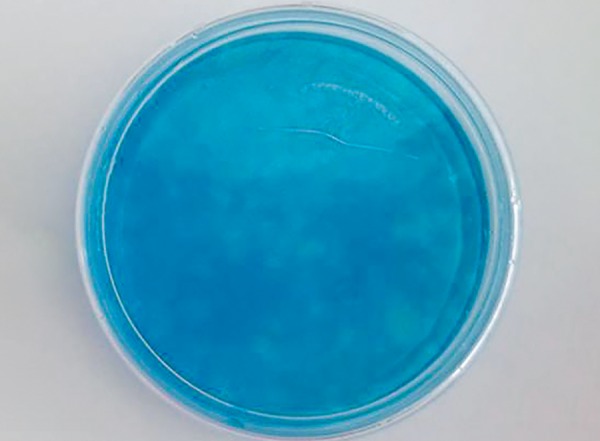
Different concentration of study groups with Mitis salivarius bacitracin agar in Petri dish

The tested bacterial strain was included Gram-positive strain *S. mutans* ATCC 25175. The study samples were inoculated on Mitis salivarius bacitracin agar medium (MSB agar), which is a highly selective medium for *S. mutans,* for counting CFU (Fig. 4). The tested bacterial inoculum of *S. mutans* of equivalent to 10^5^ CFU/mL was inoculated at 37°C for 48 hours.

**Fig. 5:**
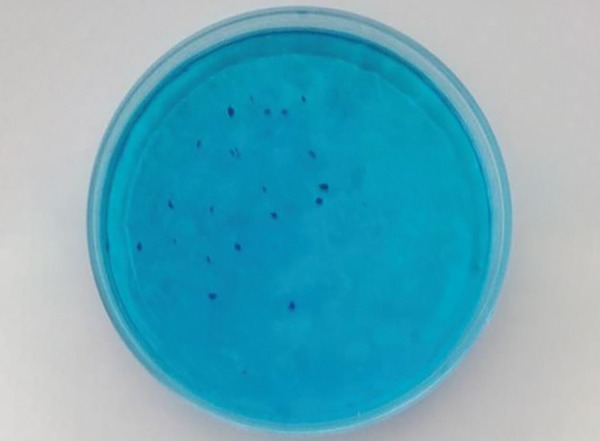
*Streptococcus mutans* in Petri dish after incubation

### Antimicrobial Activity

Grape seed extract and chlorhexidine gluconate were decided by counting *S. mutans* CFU/mL after 48-hour incubation (Fig. 5).

*Streptococcus mutans* were counted and recorded semiquantitatively as:

Grade 1 < 10^5^ CFU

Grade 2 = 10^5^-10^6^ CFU

Grade 3 > 10^6^ CFU

## STATISTICAL METHODS

The data obtained was compiled systematically, transformed from a precoded proforma to a computer, and a master table was prepared. The total data was distributed meaningfully and presented as individual tables along with graphs. Analysis of the data was made by descriptive statistics (percentages, frequency distribution) and analytical statistics using Statistical Package for the Social Sciences (SPSS) 20.0 software. Observations were subjected to statistical analysis using Krushkal-Wallis test and Wilcoxon rank sum test.

**Graph 1:**
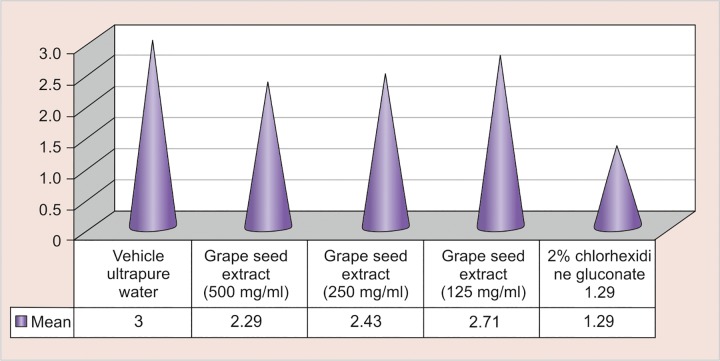
Distribution of S. *mutans* count in different study groups after 48 hours

## RESULTS

As Graph 1 shows, the distribution of *S. mutans* count was achieved after 48 hours in different groups. In vehicle ultrapure water group, maximum grade of mean value was seen which shows that it has not any bactericidal effect on *S. mutans.* While grape seed extract with 500 mg/mL concentration shows great amount of antibacterial effect, marked effect as antibacterial agent was noted with 2% chlorhexidine gluconate group.

Table 1 shows that while vehicle ultrapure water group was compared with grape seed extract at different concentrations after 48 hours, there was statistically significant difference observed with 500 and 250 mg/mL concentrations of grape seed extract; however, no statistical significance was seen with 125 mg/mL concentration of grape seed extract group. This shows that 125 mg/mL have not any significant antibacterial activity against *S. mutans* bacterial strain. Marked antibacterial activity against *S. mutans* bacteria was seen with the group of 2% chlorhexidine gluconate which shows to be a potential antibacterial agent for *S. mutans* bacteria.

**Table 1:** **Table 1:** Comparison of *Streptococcus mutans* count betweer Vehicle ultrapure water group and different concentrations of grap**e** seed extract groups (After 48 hrs.)

*Groups*		*N*		*Mean rank*		*p-value*	
Vehicle ultrapure water		7		10.00		0.007	
Grape seed extract (500 mg/mL)		7		5.00			
Vehicle ultrapure water		7		9.50		0.023	
Grape seed extract (250 mg/mL)		7		5.50			
Vehicle ultrapure water		7		8.50		0.141	
Grape seed extract (125 mg/mL)		7		6.50			
Vehicle ultrapure water		7		11.00		0.001	
2% Chlorhexidine gluconate		7		4.00			

Table 2 shows that there was no statistically significant difference seen between any concentrations of grape seed extracts. But based on mean rank value, it shows that while compared grape seed extract 500 mg/mL with 125 mg/mL, there was a marked difference between mean rank though no significant statistical difference was observed. However, based on these mean rank value 500 mg/mL concentration have more antibacterial effect than 125 mg/mL concentration of grape seed extract group.

Table 3 shows the comparison of grape seed extract of different concentrations with 2% chlorhexidine gluconate as antibacterial agent for *S. mutans.* There were statistically significant difference seen between each group of grape seed extract (different concentrations) and 2% chlorhexi-dine gluconate group. But here also, based on mean rank values, maximum significant difference was noted with 125 mg/mL concentration of grape seed extract group, which shows that this group has less antibacterial effect compared to other 250 and 500 mg/mL concentration groups of grape seed extract.

**Table 2:** **Table 2:** Comparison of *Streptococcus mutans* count between different concentrations of grape seed extracts groups (After 48 hrs.)

*Groups*		*N*		*Mean rank*		*p-value*	
Grape seed extract (500 mg/mL)		7		7.00		0.591	
Grape seed extract (250 mg/mL)		7		8.00			
Grape seed extract (500 mg/mL)		7		6.00		0.122	
Grape seed extract (125 mg/mL)		7		9.00			
Grape seed extract (250 mg/mL)		7		6.50		0.298	
Grape seed extract (125 mg/mL)		7		8.50			

**Table 3:** **Table 3:** Comparison of *Streptococcus mutans* count of different concentration grape seed extract groups with 2% chlorhexidine gluconate (After 48 hrs.)

*Groups*		*N*		*Mean rank*		*p-value*	
Grape seed extract (500 mg/mL)		7		10.29		0.006	
2% Chlorhexidine gluconate		7		4.71			
Grape seed extract (250 mg/mL)		7		10.43		0.005	
2% Chlorhexidine gluconate		7		4.57			
Grape seed extract (125 mg/mL)		7		10.71		0.002	
2% Chlorhexidine gluconate		7		4.29			

## DISCUSSION

In this study, the effect of grape seed extract *(Vitis vinifera)* with different concentrations has been evaluated against the most important bacterial strain in dental pathologies. To check their antibacterial effect, two other groups were also taken in this study, which were vehicle ultrapure water and 2% chlorhexidine gluconate.

*Streptococcus mutans* is the primary causal agent for dental caries specially in the initiation and development stages,^9^ and this microbe was first described in 1924.^10^ Grape *(Vitis vinifera)* seeds are considered rich source of polyphenolic compounds, mainly monomeric catechin and epicatechin, gallic acid and polymeric, and oligomeric procyanidins.^11^ Grape phenolics are simple molecules, such as hydroquinone, pyrocatechol, caffeic acid, ferulic acid, p-coumaric acid, gallic acid, ellagic acid, and resve-ratrol.^12^ Furthermore, grape seed extract is a rich source of diverse bioflavonoids, collectively known as grape seed proanthocyanidins extract.^13^ Polyphenols are well documented to have microbicidal activities against a huge number of pathogenic bacteria.^14,15^

The mechanism of polyphenols toxicity against microbes may be related to inhibition of hydrolytic enzymes (proteases and carbohydrolases) or other interactions to inactive microbial adhesions, cell envelope transport proteins, and nonspecific interactions with carbohydrates.^15^

In present study, chlorhexidine gluconate was taken as a positive effective agent against *S. mutans* because, as per Addy et al,^16^ chlorhexidine gluconate has significantly antibacterial effect against *S. mutans,* which was in accordance with present study. The different concentrations of grape seed extract (500, 250, and 125 mg/mL) were significantly less effective than 2% chlorhexidine gluconate.

Grape seed extract at lower concentration (125 mg/mL) was not having any significant antibacterial effect while comparing with negative control. It was supported by the study by Mirkarimi et al,^8^ which focused that grape seed extract with lower concentration has not showed any bactericidal or bacteriostatic effects against *S. mutans.*

Thimothe et al^17^ found phenolic compounds of grapes inhibit the biological activity of *S. mutans* which supported that grape seed extract with 500 and 250 mg/mL concentrations has an antibacterial effect.

In the present study, we have used the macrodilution broth method to evaluating the antimicrobial activity. This difference between methods may have an important role in making variation in results.

Therefore, based on all these findings it has been proved that grape seed extract at higher concentration acts as a natural antimicrobial compound, derived from *V. vinifera,* which has specifically inhibitory effect against *S. mutans* bacteria.

## CONCLUSION

- Grape seed extract has antibacterial effect at 250 and 500 mg/mL concentrations, but it was significantly less compared to chlorhexidine gluconate.
- Grape seed extract was not that much effective as an antibacterial agent compared to chlorhexidine gluconate.
